# Diaqua­bis­(propane-1,3-diamine)­copper(II) bis­[diamminetetra­kis­(thio­cyanato-κ*N*)chromate(III)] dimethyl sulfoxide octa­solvate

**DOI:** 10.1107/S160053681102023X

**Published:** 2011-06-04

**Authors:** Vitalina M. Nikitina, Oksana V. Nesterova, Roman I. Zubatyuk, Oleg V. Shishkin, Julia A. Rusanova

**Affiliations:** a National Taras Shevchenko University, Department of Chemistry, Volodymyrska Str. 64, 01033 Kyiv, Ukraine; b Institute for Scintillation Materials, "Institute for Single Crystals", National Academy of Sciences of Ukraine, Lenina ave. 60, Kharkov 61001, Ukraine

## Abstract

The ionic title complex, [Cu(C_3_H_10_N_2_)_2_(H_2_O)_2_][Cr(NCS)_4_(NH_3_)_2_]·8C_2_H_6_OS, consists of complex [Cu(dipr)_2_(H_2_O)_2_]^2+^ copper cations (dipr is propane-1,3-diamine), complex [Cr(NCS)_4_(NH_3_)_2_]^−^ anions and uncoord­inated solvent dimethyl sulfoxide (DMSO) mol­ecules. All the metal atoms lie on crystallographic centers of symmetry. The cations are connected to the anions through N—H⋯O hydrogen bonds between the NH_3_ mol­ecules of the anion and the water mol­ecules of the cation. The DMSO mol­ecules are involved in hydrogen-bonded linkage of the [Cr(NCS)_4_(NH_3_)_2_]^−^ anions into supra­molecular chains through bridging O atoms. A network of hydrogen bonds as well as short S⋯S contacts [3.5159 (12) and 3.4880 (12) Å] between the NCS groups of the complex anions link the mol­ecules into a three-dimensional supra­molecular network.

## Related literature

For background to direct synthesis see: Nesterov *et al.* (2004 ▶, 2006 ▶); Kovbasyuk *et al.* (1997 ▶, 1998 ▶); Vassilyeva *et al.* (1997 ▶). For the stuctures of related compexes, see: Zhang *et al.* (2001 ▶); Cucos *et al.* (2006 ▶); Cherkasova & Gorunova (2003 ▶); Kolotilov *et al.* (2010 ▶).
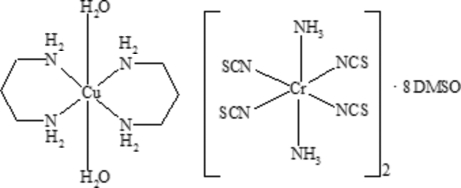


## Experimental

### 

#### Crystal data

[Cu(C_3_H_10_N_2_)_2_(H_2_O)_2_]·[Cr(NCS)_4_(NH_3_)_2_]·8(C_2_H_6_OS)
*M* *_r_* = 1509.63
Triclinic, 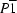

*a* = 12.2609 (11) Å
*b* = 12.2772 (12) Å
*c* = 13.8578 (12) Å
α = 72.466 (8)°
β = 89.664 (7)°
γ = 61.535 (10)°
*V* = 1724.9 (3) Å^3^
*Z* = 1
Mo *K*α radiation
μ = 1.15 mm^−1^
*T* = 100 K
0.6 × 0.4 × 0.3 mm

#### Data collection

Oxford Diffraction Xcalibur Sapphire3 diffractometer
Absorption correction: multi-scan (*CrysAlis PRO*; Oxford Diffraction, 2010 ▶) *T*_min_ = 0.58, *T*_max_ = 0.71
15229 measured reflections
8482 independent reflections
6966 reflections with *I* > 2σ(*I*)
*R*_int_ = 0.020

#### Refinement

*R*[*F* ^2^ > 2σ(*F* ^2^)] = 0.037
*wR*(*F* ^2^) = 0.097
*S* = 1.08
8482 reflections
353 parameters
H-atom parameters constrained
Δρ_max_ = 0.76 e Å^−3^
Δρ_min_ = −0.56 e Å^−3^

### 

Data collection: *CrysAlis PRO* (Oxford Diffraction, 2010 ▶); cell refinement: *CrysAlis PRO*; data reduction: *CrysAlis PRO*; program(s) used to solve structure: *SHELXTL* (Sheldrick, 2008 ▶); program(s) used to refine structure: *SHELXTL*; molecular graphics: *SHELXTL*; software used to prepare material for publication: *publCIF* (Westrip, 2010 ▶).

## Supplementary Material

Crystal structure: contains datablock(s) I, global. DOI: 10.1107/S160053681102023X/br2167sup1.cif

Structure factors: contains datablock(s) I. DOI: 10.1107/S160053681102023X/br2167Isup2.hkl

Additional supplementary materials:  crystallographic information; 3D view; checkCIF report

## Figures and Tables

**Table 1:** Hydrogen-bond geometry (Å, °)

*D*—H⋯*A*	*D*—H	H⋯*A*	*D*⋯*A*	*D*—H⋯*A*
O1—H1*E*⋯O2^i^	0.87	1.88	2.732 (2)	165
O1—H1*E*⋯S5^i^	0.87	2.87	3.5905 (18)	142
O1—H1*F*⋯O4	0.87	1.98	2.786 (2)	153
N1—H1*A*⋯O2^ii^	0.92	2.10	2.995 (3)	163
N1—H1*B*⋯S4^iii^	0.92	2.70	3.490 (2)	145
N2—H2*A*⋯O4	0.92	2.33	3.052 (2)	135
N2—H2*B*⋯O3	0.92	2.24	3.091 (3)	153
N5—H5*A*⋯O2	0.91	2.11	3.003 (2)	167
N5—H5*B*⋯O3^iv^	0.91	2.21	3.079 (2)	161
N5—H5*C*⋯O5^v^	0.91	2.09	2.966 (2)	160
N8—H8*A*⋯O1^iii^	0.91	2.08	2.956 (2)	162
N8—H8*B*⋯O5^vi^	0.91	2.19	3.054 (2)	159
N8—H8*C*⋯O3^vii^	0.91	2.10	2.981 (2)	162

Symmetry codes: (i) 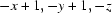; (ii) 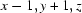; (iii) 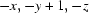; (iv) 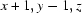; (v) 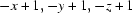; (vi) 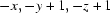; (vii) 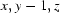.

**Fig. 1.**
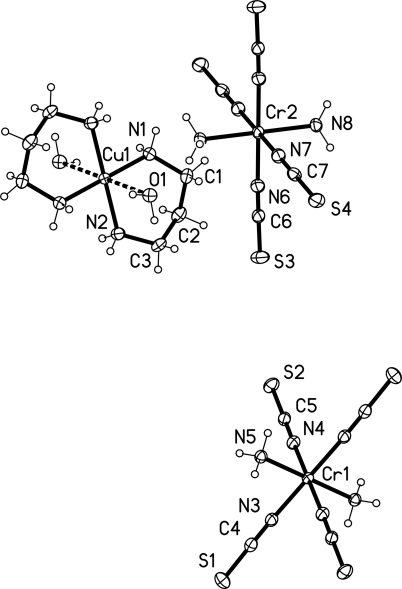
Molecular view of the title compound. DMSO molecules are omitted for clarity. Displacement ellipsoids are drawn at the 50% probability level.

**Fig. 2.**
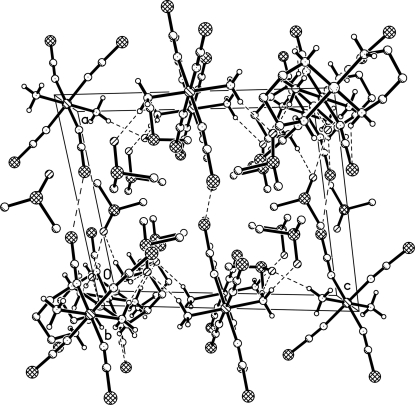
Crystal packing of the title compound along b axis.

## supplementary crystallographic information

### Comment

It has been shown that direct synthesis is an efficient method to obtained novel homo- and heterometallic complexes (Nesterov *et al.* (2004, 2006); Kovbasyuk *et al.* (1997, 1998); Vassilyeva *et al.* (1997)). Continuing our investigations in this paper we present a novel Cu/Cr heterometallic ionic complex which has been synthesized using zerovalent copper, Reinecke's salt and 1,3-propilenediamine as starting materials. As it shown on Fig. 1 Cu atoms in complex cations are in square bypiramidal coordination environment with four N atoms in equatorial position and two O atoms of the H_2_O molecules in axial position. The Cr centers are coordinated to six N atoms - four NCS-groups in equatorial position and two NH_3_ molecules in axial position. The bond distances and angles in the title molecule agree well with the corresponding bond distances and angles reported in closely related compounds (Zhang *et al.*, 2001, Cucos *et al.*, 2006; Cherkasova *et al.*, 2003; Kolotilov *et al.*, 2010). There are short interanionic S···S contacts between NCS-groups of the complex anions with the distances 3.5159 (12) and 3.4880 (12) Å whereas sum of standard Van-der-Vaals radius of the sulfur atom is 3.68 Å. The crystal packing of the title compound is presented on Fig 2.

### Experimental

For the preparation of the title compound, copper powder 0.04 g (0.63 mmol), NH_4_[Cr(NCS)_4_(NH_3_)_2_]^.^H_2_O 0.45 g (1.26 mmol), 0.11 ml (1.26 mmol) 1,3-propilenediamine (dipr), 20 ml of DMSO, were heated to 323–333 K and stirred magnetically for 15 min, until total dissolution of the copper powder was observed. Addition of a few ml of the Pr^i^OH to the cooled solution leads to precipitation within few days of the dark violet crystals suitable for X-ray analysis. They were collected by filter-suction, washed with dry Pr^i^OH and finally dried in *vacuo* at room temperature (yield: 0.59 g, 69%).

### Refinement

The structure was solved by direct methods and refined by the full-matrix least-squares technique in the anisotropic approximation for non-hydrogen atoms using the *BRUKER SHELXTL* program package. All hydrogen atoms where placed at calculated positions which were refined as 'riding' model.

### Crystal data

**Table:**

[Cu(C_3_H_10_N_2_)_2_(H_2_O)_2_]·[Cr(NCS)_4_(NH_3_)_2_]·8(C_2_H_6_OS)	*Z* = 1
*M**_r_* = 1509.63	*F*(000) = 789
Triclinic, *P*1	*D*_x_ = 1.453 Mg m^−^^3^
Hall symbol: -P 1	Mo *K*α radiation, λ = 0.71073 Å
*a* = 12.2609 (11) Å	Cell parameters from 4303 reflections
*b* = 12.2772 (12) Å	θ = 2.9–25.0°
*c* = 13.8578 (12) Å	µ = 1.15 mm^−^^1^
α = 72.466 (8)°	*T* = 100 K
β = 89.664 (7)°	Block, violet
γ = 61.535 (10)°	0.6 × 0.4 × 0.3 mm
*V* = 1724.9 (3) Å^3^	

### Data collection

**Table:**

Oxford Diffraction Xcalibur Sapphire3 diffractometer	8482 independent reflections
Radiation source: Enhance (Mo) X-ray Source	6966 reflections with *I* > 2σ(*I*)
graphite	*R*_int_ = 0.020
Detector resolution: 16.1827 pixels mm^-1^	θ_max_ = 29.9°, θ_min_ = 2.9°
ω and φ scans	*h* = −16→16
Absorption correction: multi-scan (*CrysAlis PRO*; Oxford Diffraction, 2010)	*k* = −16→15
*T*_min_ = 0.58, *T*_max_ = 0.71	*l* = −19→19
15229 measured reflections	

### Refinement

**Table:**

Refinement on *F*^2^	Primary atom site location: structure-invariant direct methods
Least-squares matrix: full	Secondary atom site location: difference Fourier map
*R*[*F*^2^ > 2σ(*F*^2^)] = 0.037	Hydrogen site location: inferred from neighbouring sites
*wR*(*F*^2^) = 0.097	H-atom parameters constrained
*S* = 1.08	*w* = 1/[σ^2^(*F*_o_^2^) + (0.0448*P*)^2^ + 1.0385*P*] where *P* = (*F*_o_^2^ + 2*F*_c_^2^)/3
8482 reflections	(Δ/σ)_max_ = 0.001
353 parameters	Δρ_max_ = 0.76 e Å^−^^3^
0 restraints	Δρ_min_ = −0.56 e Å^−^^3^

### Special details

**Table:**

Experimental. Xcalibur; Oxford Diffraction, (2010) CrysAlisPro, Oxford Diffraction (2010). Oxford Diffraction Ltd., Version 1.171.34.44 (release 25-10-2010 CrysAlis171 .NET) (compiled Oct 25 2010,18:11:34) Empirical absorption correction using spherical harmonics, implemented in SCALE3 ABSPACK scaling algorithm.
Geometry. All e.s.d.'s (except the e.s.d. in the dihedral angle between two l.s. planes) are estimated using the full covariance matrix. The cell e.s.d.'s are taken into account individually in the estimation of e.s.d.'s in distances, angles and torsion angles; correlations between e.s.d.'s in cell parameters are only used when they are defined by crystal symmetry. An approximate (isotropic) treatment of cell e.s.d.'s is used for estimating e.s.d.'s involving l.s. planes.
Refinement. Refinement of *F*^2^ against ALL reflections. The weighted *R*-factor *wR* and goodness of fit *S* are based on *F*^2^, conventional *R*-factors *R* are based on *F*, with *F* set to zero for negative *F*^2^. The threshold expression of *F*^2^ > σ(*F*^2^) is used only for calculating *R*-factors(gt) *etc*. and is not relevant to the choice of reflections for refinement. *R*-factors based on *F*^2^ are statistically about twice as large as those based on *F*, and *R*- factors based on ALL data will be even larger.

### Fractional atomic coordinates and isotropic or equivalent isotropic displacement parameters (Å^2^)

**Table:**

	*x*	*y*	*z*	*U*_iso_*/*U*_eq_	
O1	0.13010 (15)	0.77778 (15)	−0.02683 (12)	0.0259 (3)	
H1E	0.1484	0.8037	−0.0871	0.039*	
H1F	0.1991	0.7425	0.0160	0.039*	
Cu1	0.0000	1.0000	0.0000	0.01782 (9)	
Cr1	1.0000	0.0000	0.5000	0.01666 (10)	
Cr2	0.0000	0.5000	0.0000	0.01895 (11)	
S1	1.22765 (6)	0.19198 (6)	0.58717 (5)	0.03186 (14)	
S2	0.60022 (5)	0.32965 (5)	0.52979 (4)	0.02642 (13)	
S3	0.28491 (6)	0.43926 (7)	0.26932 (6)	0.03845 (16)	
S4	0.34351 (5)	0.12439 (6)	−0.04325 (5)	0.02791 (13)	
S5	0.64253 (5)	0.22031 (5)	0.19080 (4)	0.02553 (12)	
S6	0.30881 (5)	1.08392 (5)	0.23806 (4)	0.02493 (12)	
S7	0.47022 (5)	0.58296 (6)	0.11082 (4)	0.02518 (12)	
S8	0.17949 (5)	0.48082 (5)	0.67464 (4)	0.02321 (12)	
O2	0.78274 (14)	0.13337 (16)	0.19770 (12)	0.0264 (3)	
O3	0.18069 (15)	1.09692 (16)	0.22392 (13)	0.0276 (3)	
O4	0.36425 (15)	0.72167 (16)	0.05789 (12)	0.0275 (3)	
O5	0.16592 (15)	0.58928 (15)	0.71241 (12)	0.0263 (3)	
N1	−0.10137 (17)	0.93537 (17)	0.09092 (14)	0.0220 (4)	
H1A	−0.1522	1.0008	0.1162	0.026*	
H1B	−0.1535	0.9272	0.0496	0.026*	
N2	0.13971 (17)	0.92577 (18)	0.12042 (13)	0.0212 (4)	
H2A	0.2133	0.9049	0.0940	0.025*	
H2B	0.1236	0.9936	0.1440	0.025*	
N3	1.08964 (17)	0.08516 (17)	0.53719 (13)	0.0209 (4)	
N4	0.83883 (17)	0.13557 (17)	0.52307 (14)	0.0220 (4)	
N5	0.96720 (16)	0.11943 (17)	0.34908 (13)	0.0186 (3)	
H5A	0.9124	0.1123	0.3108	0.022*	
H5B	1.0410	0.0939	0.3241	0.022*	
H5C	0.9338	0.2046	0.3460	0.022*	
N6	0.12608 (17)	0.47359 (18)	0.10717 (15)	0.0242 (4)	
N7	0.13479 (17)	0.35414 (18)	−0.03865 (15)	0.0239 (4)	
N8	−0.03243 (17)	0.36323 (17)	0.10530 (14)	0.0213 (4)	
H8A	−0.0753	0.3381	0.0719	0.026*	
H8B	−0.0786	0.3996	0.1501	0.026*	
H8C	0.0423	0.2917	0.1403	0.026*	
C1	−0.0366 (2)	0.8106 (2)	0.17926 (17)	0.0254 (5)	
H1C	0.0134	0.7365	0.1541	0.031*	
H1D	−0.0998	0.7942	0.2157	0.031*	
C2	0.0489 (2)	0.8171 (2)	0.25237 (17)	0.0266 (5)	
H2C	0.0741	0.7434	0.3179	0.032*	
H2D	0.0018	0.9003	0.2669	0.032*	
C3	0.1658 (2)	0.8099 (2)	0.21183 (16)	0.0256 (5)	
H3A	0.2224	0.8027	0.2669	0.031*	
H3B	0.2103	0.7291	0.1935	0.031*	
C4	1.1462 (2)	0.1305 (2)	0.55823 (16)	0.0215 (4)	
C5	0.7391 (2)	0.2164 (2)	0.52692 (16)	0.0207 (4)	
C6	0.1937 (2)	0.4591 (2)	0.17413 (18)	0.0240 (4)	
C7	0.2218 (2)	0.2583 (2)	−0.04054 (16)	0.0219 (4)	
C8	0.5722 (2)	0.1351 (3)	0.1611 (2)	0.0333 (5)	
H8D	0.5821	0.1333	0.0913	0.050*	
H8E	0.4826	0.1801	0.1659	0.050*	
H8F	0.6131	0.0447	0.2098	0.050*	
C9	0.6182 (3)	0.1991 (3)	0.3196 (2)	0.0557 (10)	
H9A	0.6514	0.1053	0.3578	0.084*	
H9B	0.5280	0.2480	0.3214	0.084*	
H9C	0.6619	0.2323	0.3511	0.084*	
C10	0.4189 (2)	0.9225 (2)	0.2392 (2)	0.0311 (5)	
H10A	0.4043	0.8578	0.2903	0.047*	
H10B	0.5046	0.9045	0.2568	0.047*	
H10C	0.4083	0.9167	0.1712	0.047*	
C11	0.3450 (3)	1.0572 (3)	0.3702 (2)	0.0402 (6)	
H11A	0.2926	1.1390	0.3842	0.060*	
H11B	0.4340	1.0294	0.3870	0.060*	
H11C	0.3285	0.9883	0.4122	0.060*	
C12	0.6073 (2)	0.5762 (3)	0.0626 (2)	0.0329 (5)	
H12A	0.6020	0.5784	−0.0086	0.049*	
H12B	0.6818	0.4946	0.1046	0.049*	
H12C	0.6135	0.6520	0.0652	0.049*	
C13	0.5117 (2)	0.5740 (3)	0.23673 (19)	0.0354 (6)	
H13A	0.5284	0.6457	0.2328	0.053*	
H13B	0.5873	0.4892	0.2718	0.053*	
H13C	0.4423	0.5824	0.2750	0.053*	
C14	0.0292 (2)	0.4947 (3)	0.6669 (2)	0.0331 (5)	
H14A	0.0074	0.4758	0.7358	0.050*	
H14B	0.0302	0.4317	0.6367	0.050*	
H14C	−0.0334	0.5844	0.6239	0.050*	
C15	0.1823 (3)	0.5356 (3)	0.54062 (19)	0.0348 (6)	
H15A	0.1095	0.6237	0.5080	0.052*	
H15B	0.1791	0.4744	0.5104	0.052*	
H15C	0.2599	0.5389	0.5298	0.052*	

### Atomic displacement parameters (Å^2^)

**Table:**

	*U*^11^	*U*^22^	*U*^33^	*U*^12^	*U*^13^	*U*^23^
O1	0.0239 (8)	0.0248 (8)	0.0277 (8)	−0.0124 (7)	0.0004 (6)	−0.0066 (6)
Cu1	0.01624 (17)	0.01650 (17)	0.01893 (17)	−0.00831 (14)	0.00019 (13)	−0.00339 (13)
Cr1	0.0145 (2)	0.0143 (2)	0.0189 (2)	−0.00612 (17)	0.00164 (17)	−0.00447 (17)
Cr2	0.0162 (2)	0.0144 (2)	0.0248 (2)	−0.00683 (18)	0.00464 (18)	−0.00617 (18)
S1	0.0354 (3)	0.0315 (3)	0.0385 (3)	−0.0230 (3)	0.0043 (3)	−0.0136 (3)
S2	0.0196 (3)	0.0210 (3)	0.0285 (3)	−0.0031 (2)	0.0046 (2)	−0.0073 (2)
S3	0.0249 (3)	0.0358 (3)	0.0474 (4)	−0.0128 (3)	−0.0097 (3)	−0.0085 (3)
S4	0.0189 (3)	0.0210 (3)	0.0415 (3)	−0.0054 (2)	0.0044 (2)	−0.0154 (2)
S5	0.0205 (3)	0.0208 (3)	0.0282 (3)	−0.0047 (2)	−0.0019 (2)	−0.0087 (2)
S6	0.0225 (3)	0.0225 (3)	0.0303 (3)	−0.0126 (2)	0.0066 (2)	−0.0071 (2)
S7	0.0186 (3)	0.0235 (3)	0.0323 (3)	−0.0096 (2)	0.0007 (2)	−0.0095 (2)
S8	0.0230 (3)	0.0177 (2)	0.0258 (3)	−0.0086 (2)	0.0018 (2)	−0.0060 (2)
O2	0.0186 (8)	0.0266 (8)	0.0288 (8)	−0.0080 (6)	−0.0002 (6)	−0.0083 (7)
O3	0.0203 (8)	0.0248 (8)	0.0363 (9)	−0.0109 (7)	0.0059 (7)	−0.0093 (7)
O4	0.0193 (8)	0.0240 (8)	0.0304 (8)	−0.0060 (6)	0.0000 (6)	−0.0061 (6)
O5	0.0292 (8)	0.0210 (8)	0.0282 (8)	−0.0115 (7)	0.0020 (7)	−0.0093 (6)
N1	0.0193 (9)	0.0189 (9)	0.0257 (9)	−0.0096 (7)	0.0015 (7)	−0.0046 (7)
N2	0.0185 (8)	0.0228 (9)	0.0208 (8)	−0.0095 (7)	0.0018 (7)	−0.0065 (7)
N3	0.0192 (9)	0.0166 (8)	0.0228 (8)	−0.0069 (7)	0.0025 (7)	−0.0048 (7)
N4	0.0202 (9)	0.0183 (9)	0.0233 (9)	−0.0078 (7)	0.0020 (7)	−0.0046 (7)
N5	0.0183 (8)	0.0173 (8)	0.0191 (8)	−0.0081 (7)	0.0033 (7)	−0.0059 (6)
N6	0.0192 (9)	0.0208 (9)	0.0308 (10)	−0.0094 (7)	0.0033 (8)	−0.0076 (8)
N7	0.0201 (9)	0.0198 (9)	0.0306 (9)	−0.0089 (7)	0.0064 (7)	−0.0085 (7)
N8	0.0205 (9)	0.0168 (8)	0.0269 (9)	−0.0100 (7)	0.0041 (7)	−0.0067 (7)
C1	0.0270 (11)	0.0196 (10)	0.0251 (10)	−0.0115 (9)	0.0022 (9)	−0.0018 (8)
C2	0.0279 (12)	0.0238 (11)	0.0197 (10)	−0.0085 (9)	0.0026 (9)	−0.0041 (8)
C3	0.0235 (11)	0.0232 (11)	0.0197 (10)	−0.0073 (9)	−0.0039 (8)	−0.0012 (8)
C4	0.0215 (10)	0.0176 (10)	0.0212 (10)	−0.0078 (8)	0.0026 (8)	−0.0045 (8)
C5	0.0222 (10)	0.0176 (10)	0.0204 (9)	−0.0096 (8)	0.0020 (8)	−0.0046 (8)
C6	0.0176 (10)	0.0173 (10)	0.0324 (11)	−0.0073 (8)	0.0041 (9)	−0.0046 (9)
C7	0.0194 (10)	0.0235 (11)	0.0267 (10)	−0.0122 (9)	0.0067 (8)	−0.0110 (9)
C8	0.0254 (12)	0.0302 (12)	0.0427 (14)	−0.0155 (10)	0.0030 (10)	−0.0075 (11)
C9	0.0343 (15)	0.064 (2)	0.0323 (14)	0.0075 (14)	0.0003 (12)	−0.0237 (14)
C10	0.0228 (11)	0.0241 (11)	0.0393 (13)	−0.0085 (9)	0.0057 (10)	−0.0070 (10)
C11	0.0467 (16)	0.0494 (17)	0.0368 (14)	−0.0317 (14)	0.0075 (12)	−0.0169 (12)
C12	0.0197 (11)	0.0330 (13)	0.0436 (14)	−0.0112 (10)	0.0056 (10)	−0.0133 (11)
C13	0.0267 (12)	0.0367 (14)	0.0314 (12)	−0.0080 (11)	−0.0039 (10)	−0.0101 (11)
C14	0.0312 (13)	0.0326 (13)	0.0399 (13)	−0.0195 (11)	0.0069 (11)	−0.0119 (11)
C15	0.0510 (16)	0.0367 (14)	0.0314 (12)	−0.0301 (13)	0.0165 (11)	−0.0166 (11)

### Geometric parameters (Å, °)

**Table:**

O1—H1E	0.8692	N4—C5	1.165 (3)
O1—H1F	0.8704	N5—H5A	0.9100
Cu1—O1	2.5655 (16)	N5—H5B	0.9100
Cu1—N1	2.0202 (18)	N5—H5C	0.9100
Cu1—N1^i^	2.0202 (18)	N6—C6	1.162 (3)
Cu1—N2^i^	2.0442 (17)	N7—C7	1.159 (3)
Cu1—N2	2.0442 (17)	N8—H8A	0.9100
Cr1—N4	1.9888 (18)	N8—H8B	0.9100
Cr1—N4^ii^	1.9888 (18)	N8—H8C	0.9100
Cr1—N3^ii^	1.9983 (19)	C1—C2	1.508 (3)
Cr1—N3	1.9983 (19)	C1—H1C	0.9900
Cr1—N5	2.0702 (17)	C1—H1D	0.9900
Cr1—N5^ii^	2.0702 (17)	C2—C3	1.509 (3)
Cr2—N6	1.9898 (19)	C2—H2C	0.9900
Cr2—N6^iii^	1.9898 (19)	C2—H2D	0.9900
Cr2—N7^iii^	1.9928 (18)	C3—H3A	0.9900
Cr2—N7	1.9928 (19)	C3—H3B	0.9900
Cr2—N8	2.0640 (17)	C8—H8D	0.9800
Cr2—N8^iii^	2.0641 (17)	C8—H8E	0.9800
S1—C4	1.623 (2)	C8—H8F	0.9800
S2—C5	1.615 (2)	C9—H9A	0.9800
S3—C6	1.617 (2)	C9—H9B	0.9800
S4—C7	1.622 (2)	C9—H9C	0.9800
S5—O2	1.5149 (16)	C10—H10A	0.9800
S5—C8	1.769 (3)	C10—H10B	0.9800
S5—C9	1.770 (3)	C10—H10C	0.9800
S6—O3	1.5090 (17)	C11—H11A	0.9800
S6—C11	1.780 (3)	C11—H11B	0.9800
S6—C10	1.783 (2)	C11—H11C	0.9800
S7—O4	1.5086 (16)	C12—H12A	0.9800
S7—C13	1.777 (3)	C12—H12B	0.9800
S7—C12	1.779 (2)	C12—H12C	0.9800
S8—O5	1.5108 (17)	C13—H13A	0.9800
S8—C14	1.769 (3)	C13—H13B	0.9800
S8—C15	1.781 (2)	C13—H13C	0.9800
N1—C1	1.483 (3)	C14—H14A	0.9800
N1—H1A	0.9200	C14—H14B	0.9800
N1—H1B	0.9200	C14—H14C	0.9800
N2—C3	1.489 (3)	C15—H15A	0.9800
N2—H2A	0.9200	C15—H15B	0.9800
N2—H2B	0.9200	C15—H15C	0.9800
N3—C4	1.159 (3)		
			
H1E—O1—H1F	105.3	Cr2—N8—H8B	109.5
N1—Cu1—N1^i^	180.00 (11)	H8A—N8—H8B	109.5
N1—Cu1—N2^i^	87.84 (7)	Cr2—N8—H8C	109.5
N1^i^—Cu1—N2^i^	92.16 (7)	H8A—N8—H8C	109.5
N1—Cu1—N2	92.16 (7)	H8B—N8—H8C	109.5
N1^i^—Cu1—N2	87.84 (7)	N1—C1—C2	110.69 (19)
N2^i^—Cu1—N2	180.0	N1—C1—H1C	109.5
N1—Cu1—O1	92.27 (6)	C2—C1—H1C	109.5
N1^i^—Cu1—O1	87.73 (6)	N1—C1—H1D	109.5
N2^i^—Cu1—O1	94.59 (6)	C2—C1—H1D	109.5
N2—Cu1—O1	85.41 (6)	H1C—C1—H1D	108.1
N4—Cr1—N4^ii^	180.00 (10)	C1—C2—C3	113.34 (19)
N4—Cr1—N3^ii^	89.65 (8)	C1—C2—H2C	108.9
N4^ii^—Cr1—N3^ii^	90.35 (8)	C3—C2—H2C	108.9
N4—Cr1—N3	90.35 (8)	C1—C2—H2D	108.9
N4^ii^—Cr1—N3	89.65 (8)	C3—C2—H2D	108.9
N3^ii^—Cr1—N3	180.00 (6)	H2C—C2—H2D	107.7
N4—Cr1—N5	90.24 (7)	N2—C3—C2	113.53 (18)
N4^ii^—Cr1—N5	89.76 (7)	N2—C3—H3A	108.9
N3^ii^—Cr1—N5	91.87 (7)	C2—C3—H3A	108.9
N3—Cr1—N5	88.13 (7)	N2—C3—H3B	108.9
N4—Cr1—N5^ii^	89.76 (7)	C2—C3—H3B	108.9
N4^ii^—Cr1—N5^ii^	90.24 (7)	H3A—C3—H3B	107.7
N3^ii^—Cr1—N5^ii^	88.13 (7)	N3—C4—S1	178.9 (2)
N3—Cr1—N5^ii^	91.87 (7)	N4—C5—S2	178.86 (19)
N5—Cr1—N5^ii^	180.000 (1)	N6—C6—S3	178.4 (2)
N6—Cr2—N6^iii^	180.0	N7—C7—S4	179.9 (3)
N6—Cr2—N7^iii^	90.37 (8)	S5—C8—H8D	109.5
N6^iii^—Cr2—N7^iii^	89.63 (8)	S5—C8—H8E	109.5
N6—Cr2—N7	89.63 (8)	H8D—C8—H8E	109.5
N6^iii^—Cr2—N7	90.37 (8)	S5—C8—H8F	109.5
N7^iii^—Cr2—N7	180.00 (10)	H8D—C8—H8F	109.5
N6—Cr2—N8	89.48 (8)	H8E—C8—H8F	109.5
N6^iii^—Cr2—N8	90.52 (8)	S5—C9—H9A	109.5
N7^iii^—Cr2—N8	91.17 (7)	S5—C9—H9B	109.5
N7—Cr2—N8	88.83 (7)	H9A—C9—H9B	109.5
N6—Cr2—N8^iii^	90.52 (8)	S5—C9—H9C	109.5
N6^iii^—Cr2—N8^iii^	89.48 (8)	H9A—C9—H9C	109.5
N7^iii^—Cr2—N8^iii^	88.83 (7)	H9B—C9—H9C	109.5
N7—Cr2—N8^iii^	91.17 (7)	S6—C10—H10A	109.5
N8—Cr2—N8^iii^	180.00 (14)	S6—C10—H10B	109.5
O2—S5—C8	105.70 (11)	H10A—C10—H10B	109.5
O2—S5—C9	104.89 (11)	S6—C10—H10C	109.5
C8—S5—C9	98.98 (17)	H10A—C10—H10C	109.5
O3—S6—C11	105.98 (12)	H10B—C10—H10C	109.5
O3—S6—C10	105.91 (11)	S6—C11—H11A	109.5
C11—S6—C10	97.80 (13)	S6—C11—H11B	109.5
O4—S7—C13	106.08 (11)	H11A—C11—H11B	109.5
O4—S7—C12	106.13 (11)	S6—C11—H11C	109.5
C13—S7—C12	97.55 (13)	H11A—C11—H11C	109.5
O5—S8—C14	105.80 (11)	H11B—C11—H11C	109.5
O5—S8—C15	106.19 (11)	S7—C12—H12A	109.5
C14—S8—C15	97.93 (13)	S7—C12—H12B	109.5
C1—N1—Cu1	120.07 (14)	H12A—C12—H12B	109.5
C1—N1—H1A	107.3	S7—C12—H12C	109.5
Cu1—N1—H1A	107.3	H12A—C12—H12C	109.5
C1—N1—H1B	107.3	H12B—C12—H12C	109.5
Cu1—N1—H1B	107.3	S7—C13—H13A	109.5
H1A—N1—H1B	106.9	S7—C13—H13B	109.5
C3—N2—Cu1	121.96 (14)	H13A—C13—H13B	109.5
C3—N2—H2A	106.8	S7—C13—H13C	109.5
Cu1—N2—H2A	106.8	H13A—C13—H13C	109.5
C3—N2—H2B	106.8	H13B—C13—H13C	109.5
Cu1—N2—H2B	106.8	S8—C14—H14A	109.5
H2A—N2—H2B	106.7	S8—C14—H14B	109.5
C4—N3—Cr1	177.22 (18)	H14A—C14—H14B	109.5
C5—N4—Cr1	173.03 (18)	S8—C14—H14C	109.5
Cr1—N5—H5A	109.5	H14A—C14—H14C	109.5
Cr1—N5—H5B	109.5	H14B—C14—H14C	109.5
H5A—N5—H5B	109.5	S8—C15—H15A	109.5
Cr1—N5—H5C	109.5	S8—C15—H15B	109.5
H5A—N5—H5C	109.5	H15A—C15—H15B	109.5
H5B—N5—H5C	109.5	S8—C15—H15C	109.5
C6—N6—Cr2	175.38 (19)	H15A—C15—H15C	109.5
C7—N7—Cr2	166.49 (18)	H15B—C15—H15C	109.5
Cr2—N8—H8A	109.5		

Symmetry codes: (i) −*x*, −*y*+2, −*z*; (ii) −*x*+2, −*y*, −*z*+1; (iii) −*x*, −*y*+1, −*z*.

### Hydrogen-bond geometry (Å, °)

**Table:**

*D*—H···*A*	*D*—H	H···*A*	*D*···*A*	*D*—H···*A*
O1—H1E···O2^iv^	0.87	1.88	2.732 (2)	165
O1—H1E···S5^iv^	0.87	2.87	3.5905 (18)	142
O1—H1F···O4	0.87	1.98	2.786 (2)	153
N1—H1A···O2^v^	0.92	2.10	2.995 (3)	163
N1—H1B···S4^iii^	0.92	2.70	3.490 (2)	145
N2—H2A···O4	0.92	2.33	3.052 (2)	135
N2—H2B···O3	0.92	2.24	3.091 (3)	153
N5—H5A···O2	0.91	2.11	3.003 (2)	167
N5—H5B···O3^vi^	0.91	2.21	3.079 (2)	161
N5—H5C···O5^vii^	0.91	2.09	2.966 (2)	160
N8—H8A···O1^iii^	0.91	2.08	2.956 (2)	162
N8—H8B···O5^viii^	0.91	2.19	3.054 (2)	159
N8—H8C···O3^ix^	0.91	2.10	2.981 (2)	162

Symmetry codes: (iv) −*x*+1, −*y*+1, −*z*; (v) *x*−1, *y*+1, *z*; (iii) −*x*, −*y*+1, −*z*; (vi) *x*+1, *y*−1, *z*; (vii) −*x*+1, −*y*+1, −*z*+1; (viii) −*x*, −*y*+1, −*z*+1; (ix) *x*, *y*−1, *z*.
